# Supplementary material for: Analysis of genetically determined gene expression suggests role of inflammatory processes in exfoliation syndrome
Source: BMC Genomics. 2023 Feb 16;24:75. doi: 10.1186/s12864-023-09179-7 (PMC9936777; doi:10.1186/s12864-023-09179-7)

Supplemental Information

Analysis of Genetically Determined Gene Expression Suggests Role of Inflammatory Processes in Exfoliation Syndrome

Jibril B. Hirbo,[1,4*] Francesca Pasutto,[2] Eric R. Gamazon,[1,4,5] Patrick Evans,[1] Priyanka Pawar,[3] Daniel Berner,[7] Julia Sealock,[1] Ran Tao,[6] Peter S. Straub,[1] Anuar I. Konkashbaev,[1] Max A. Breyer,[1] Ursula Schlötzer-Schrehardt,[7] André Reis,[2] Milam A. Brantley Jr.,[3] Chiea C. Khor,[8] Karen M. Joos,[3] Nancy J. Cox,[1,4]

Corresponding Author: Jibril Hirbo* *jibril.hirbo@vumc.org*

Additional data for the study from BioVU

Our study leveraged (a) global GWAS data consisting of 13,620 XFS cases and 109,837 controls and (b) individual-level genetic data from two independent datasets comprising 4,127 cases and 9,075 controls. PrediXcan analysis in European ancestry individuals included additional data from Vanderbilt University Medical Center (VUMC) biobank. VUMC has built the largest academic medical center-based biobank (BioVU) in the USA that contains DNA extracted from discarded blood collected during routine clinical testing and linked to de-identified electronic health record (EHR) information.[1] All DNA samples collected since 2014 have patient consent for research purposes. This study was approved by the Institutional Review Board at Vanderbilt University. Workflows for the detailed European subsets of multi-ethnic global data. is in the Supplementary section of the study, and sample selection from BioVU data in **Suppl. Fig. S1** and description of subject selection below.

Subject Selection from BioVU

Subjects (n=144,017) with an ophthalmology examination code (CPT = 92002, 92004, 92012, or 92014) were initially separated by ICD-9 and/or ICD-10 codes for 1) exfoliation syndrome/pseudo-exfoliation with or without glaucoma (366.11, H25.89/365.52, H40.14XX) and 2) controls with no glaucoma or optic nerve disease (n=53,919 all $\geq$ 45 years old excluding codes 366.11, H25.89, 365.XX, H40.XXXX,/ 377.XX, H47.XXXX). The groups were then reduced to include only subjects with genotyping data: 1) XFS - 41 cases, 2) controls – 7,105. To confirm the diagnosis, the de-identified electronic medical records of the XFS subjects were then reviewed. Available records included the problem list, stating exfoliation syndrome or pseudo-exfoliation; medication lists; clinical notes, including detailed ophthalmic examinations;

ophthalmic operative notes; correspondence letters; and discharge summaries. Additional high-level confirmation included an anterior segment examination description of exfoliation material deposits, or pupillary transillumination defects.

Only those BioVU subjects (n=41) who had XFS consistently reported in any of the above categories by the treating physicians were included in the study. For the BioVU data, any subject coded for XFS without supporting confirmation or who had contradictory information in the medical record, was excluded to minimize inclusion errors. Additional information, including reported peripheral vision loss, intraocular pressure, glaucomatous optic nerve appearance, glaucoma surgeries and use of glaucoma medications, was reviewed and noted.

Genotyping

The BioVU cases and controls were genotyped on five different Illumina genotyping arrays; Human660W-Quad, HumanOmni1-Quad, Infinium Omni5-4, OmniExpress-8v1-2-B (jointly as Others) and Infinium Multi-Ethnic Global-8 (MEGA). The data were processed using established GWAS quality control procedures[8], and imputed on the Michigan Imputation server using 1000Genomes phase 3 v.5 release (October 2014) mixed ancestry panel using MaCH (v. 1.0.2) and Minimac (release 6/27/2017) **(Suppl. Fig. S2)**. For these samples, genotypes for a total of 290,778 SNPs (Others) and 1,833,203 SNPs (MEGA) were extended by imputation from the experimental genotypes and after filtering (at imputation quality score (r^2) ≥ 0.7) a total of 8,421,562 SNPs (Others) and 24,188,561 SNPs (MEGA) remained for analysis.

Description of Supplemental Data

Supplemental Data include 7 figures: 1-5,6a, 6b, and 21 tables: 1-6,7a-7g, 9a-9e, 10a, 10b

Suppl. Figure S1: Workflow for selecting XFS cases and controls in BioVU dataset
[1]Procedure code indicating that individuals had eye exam or care at Vanderbilt Eye Institute
[2]Patients genotyped on Illumina 660W, Illumina 1M, Illumina Omni-Quad, Illumina Omni5-Quad & Human Omni-Express-24v1-0-B, excluding individuals that have genotyped data on Illumina mega-x-array
[3]Based on genetic ancestry using PCA

Suppl. Figure S2: Pre-analysis workflow for quality controls of Mega-array dataset

Suppl. Figure S3: Manhattan plot for GWAS meta-analysis and PrediXcan analysis of European Pseudo-exfoliation syndrome (XFS) data; a) The lower half of the plot is for the XFS meta-analysis summary statistics data Aung et al., 2017, while the upper half of the plot shows results from PrediXcan analysis for 48 GTEx tissues. On the X axis is plot of variant/gene associations along the chromosomes, while Y axis represent the significance levels for the associations. The legend for PrediXcan analysis on the 48 GTEx tissues, a color for each tissue, is on the right. For both plots the blue dotted line is the "suggestive" genome-wide significant threshold ($p<1e-4$), while the red line is the genome-wide significant threshold. On the lower plot, the gene labels are for genes reported/mapped to genome-wide significant signals in GWAS result, while in the upper plot is for genes that are associated at genome-wide significant threshold. For genes associated with XFS at genome-wide threshold in more than one tissues, only the tissue with lowest p-value is labeled.

Suppl. Figure S4: Correlation between association strength chromosome 15q22-25 genes with measured gene expression versus LOXL1 and STOML1.

Suppl. Figure S5: Plot of expression levels of genes implicated in XFS in different Eye tissues. The plot was generated using data obtained from Ocular tissue Database.

Suppl. Figure S6a: Comorbidity analysis XFS with other phecodes in BioVU European ancestry individuals (n ~900k)

Suppl. Figure 6b: Plot of regression analysis of polygenic risk generated from Multi-ethnic ancestry summary statistics against European ancestry individuals in BioVU (n ~62k).

Suppl. Table S1: DNA Oligonucleotides used for validation analysis

Suppl. Table S2: Genes Associated with XFS in multi-ethnic global dataset: Results of single tissue analysis of global multi-ethnic summary statistics for XFS from Aung et al., 2017

Suppl. Table S3: Genes Associated with XFS in multi-ethnic global dataset: Results of cross- tissue analysis of global multi-ethnic summary statistics for XFS from Aung et al., 2017

Suppl. Table S4: Genes Associated with XFS in Europeans: Results of single tissue analysis of European summary statistics for XFS from a Pasutto et al., 2017 and BioVU data

Suppl. Table S5: Genes Associated with XFS in Europeans: Results of cross-tissue analysis of European summary statistics for XFS from a Pasutto et al., 2017 and BioVU data

Suppl. Table S6: Genetic Associations replicated in Europeans: column 3-5 is for cross-tissues analysis, the third column (pvalue_multitissue) is p-value of association in cross-tissue analysis in multi-ethnic ancestry data, fourth column (n) is number of total tissues involved in cross-tissue analysis, fifth column is (n_indep) number of tissues with independent gene expressions. Columns 6-8 represent lowest p-value for the gene in the multiethnic data, the effect size in the lowest reported p-value and tissue in which the association is with XFS, respectively. Column 9 and 10 represent lowest p-value for the gene in the Europeans and tissue in which the association is with XFS, respectively.

Suppl. Table S7a: Correlation between association signal in chr15q22-25 and GTEx gene expression correlation with *LOXL1* and *STOML1*

Suppl. Table S7b: Effects of excluding rs1048661 and rs3825942 variants from Prediction models on chr15q22-25 gene associations with XFS

Suppl. Table S7c: Variants in LD with rs3825942 with diminishing effect on associations with XFS in the Prediction models for chr15q22-25 genes

Suppl. Table S7d: LD with rs3825942 correlation for variants with diminishing effect on associations with XFS in the Prediction models for chr15q22-25 genes with magnitude of shift in associations.

Suppl. Table S7e: Effect of excluding variants in LD with STOML1/LOXL1 or shared with others genes in from chr15q22-25

Suppl. Table S7f: Variants in LD with STOML1/LOXL1 with diminishing effect on associations with XFS in the Prediction models for chr15q22-25 genes

Suppl. Table S7g: Lack of LD with rs3825942 for variants in LD with STOML1/LOXL1 with diminishing effect on associations with XFS in the Prediction models for chr15q22-25 genes. Genes in blue are those that had effect reversal in South African populations in Aung et al.

Suppl. Table S8: Prioritization of genes that are associated with XFS: Each gene pair was checked if they had correlated predicted and expressed gene expressions, and LD checked for variants included in prediction models

Suppl. Table 9a: Enrichment analysis of genes that are associated to XFS at genome-wide significance levels (p<2.02e-7) (Enrichr)

Suppl. Table 9b: Enrichment analysis of genes that are associated to XFS at nominal significance levels (p<0.05) (Enrichr & Reactome)

Suppl. Table 9c: Enrichment analysis of genes that are associated to XFS at nominal significance levels (p<0.05) excluding HLA genes (Enrichr)

Suppl. Table 9d: Enrichment analysis of genes that are associated to XFS at nominal significance levels (p<0.05) (GSEA)

Suppl. Table 9e: Enrichment analysis of genes that are associated to XFS at nominal significance levels (p<0.05) and predicted to be downregulated (Enrichr)

Suppl. Table 10a: Traits in the Electronic Health records that are comorbid with XFS in BioVU biobank

Suppl. Table 10b: Traits in the Electronic Health records that individuals in BioVU biobank with XFS genetic liability are at increased risk for

Supplementary Table S1: DNA Oligonucleotides used in this study.

Primer Name	Sequence (5'-3')	Product (bp)
NEO1-F	CACGGAACTCAAAGGGCATG	119
NEO1-R	GCTTCCTTTCCCTCCAGACC	
CD276-F	GGCTTTCGTGTGCTGGAGAA	119
CD276-R	CAGAGTGTTTCAGAGGCTGC	
INSYN1-F1	GGCTCCAGTTCAAAGTCGAA	80
INSYN1-R1:	CAAGGAGGTGGCCAAGGAG	
LOXL1-F	CGTGGGCAGCGTGTAC	147
LOXL1-R	CTGGCCAGACACTTCTCCTC	
STOML1-F	GCCCTGGTCACATCGTTGAT	141
STOML1-R	AGACCTGAACACAGCCACAC	
UBL7-F	TGGGATCAGCGAAGACAGAG	158
UBL7-R	GCACAGCAGCTCCTCTTACA	
ARID3B-F	CCAAAGATGCTTCCAAGGCC	112
ARID3B-R	TCTGCATCATCACTCCAGGC	
SCAMP2-F	CAAAGGAAGGCACAGGGAGT	186
SCAMP2-R	AGAGATCCCTGCCGACTACC	
GAPDH-F	AAGGTCGGAGTCAACGGATTTGG	194
GAPDH-R	ATGACAAGCTTCCCGTTCTCAGC	

Supplementary Table S8: Prioritization of genes that are associated with XFS

Tissues	Significant Genes[1]	Conditional Analysis		Correlated expression ($r^2 \geq \pm 0.2$)	Correlated predicted expression ($r^2 \geq \pm 0.1$)	LD between SNPs in Prediction models (r2>0.3)+	LD between SNPs in Prediction models (r2>0.3)y	Prediction models share variants
Lung	Genes (pvalue)	*LOXL1*	*STOML1*					
	LOXL1 (8.75e-10)	SCAMP2 (2.73e-28)	SCAMP2 (1.01e-31)	Yes	No	No	LOXL1 (q)	Yes
	STOML1 (6.60e-9)	INSYN1 (1.91e-10)		Yes	Yes	No		Yes
Brain Cerebellum	Genes (pvalue)	*STOML1*	*ISLR*	No	No	Yes		Yes
	STOML1 (1.48E-11)	NS	NS					
	ISLR (3.65E-08)						ISLR (p, q)	
Heart left ventricle	Genes (pvalue)	*MPI*	*STOML1*	Yes	No	Yes		Yes
	MPI (7.11E-17)	UBL7 (1.56E-16)	UBL7 (3.89E-10)	Yes	Yes	Yes	MPI (p)	No
	STOML1 (3.98E-13)							
Heart atrial appendage	Genes (pvalue)	*STOML1*	*INSYN1*	Yes	No	No		Yes
	INSYN1 (4.20E-16)	NS	STOML1 (2.57E-12)					
	STOML1 (3.72E-10)							
Colon transverse	Genes (pvalue)	*STOML1*		No	No	Yes		No
	LMANL1 (5.61e-10)	NS						
	STOML1 (5.54e-9)						STOML1 (p, q)	
Esophagus_Muscularis	Genes (pvalue)	*CYP1A1*	*UBL7*	No	No	Yes		Yes
	CYP1A1 (9.12E-11)	STOML1 (2.88E-97)	STOML1 (2.02E-98)	No	No	Yes	STOML1 (q)	Yes
	UBL7 (2.27E-08)	NEO1 (3.57E-09)	NEO1 (5.34E-09)	No	Yes	No		No

[1]Genes associated with XFS in European dataset at genome-wide threshold (>2.02e-7)

+LD between variants in model of gene pairs

yLD between variants in a model of gene and one of the coding variants (p) rs1048661- rs4886776 (q) rs3825942

NS Not significantly associated at genome-wide threshold

Suppl. Figure S1: Workflow for selection of XFS cases and controls in BioVU dataset

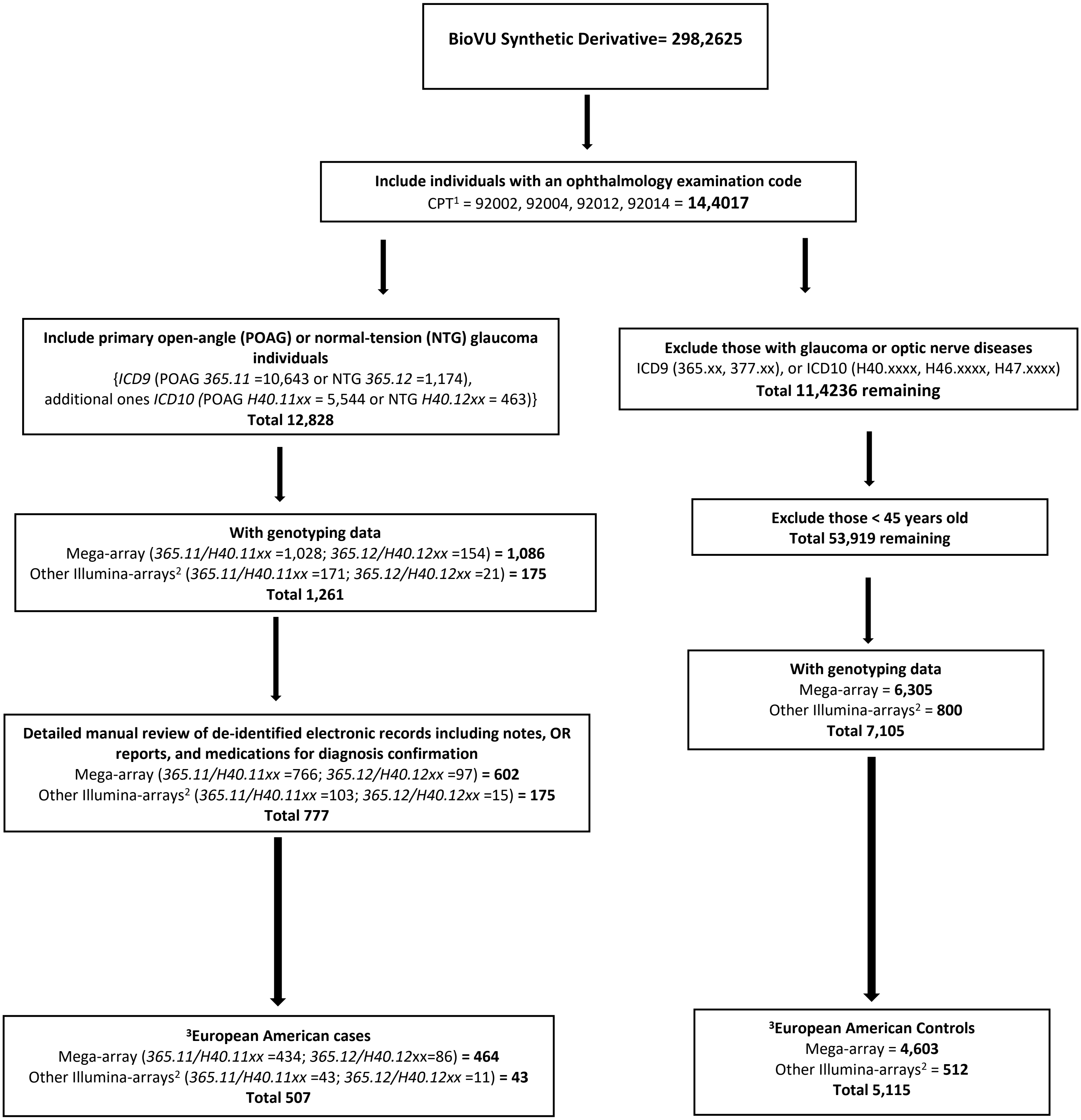

1) Procedure code indicating that individuals had eye exam or care at Vanderbilt Eye Institute
2) Patients genotyped on Illumina 660W, Illumina 1M, Illumina Omni-Quad, Illumina Omni5-Quad & Human Omni-Express-24v1-0-B, excluding individuals that have genotyped data on Illumina mega-x-array
3) Based on genetic ancestry using PCA

Suppl. Figure S2: Pre-analysis workflow for quality controls of Mega-array dataset

Mega-array Data from BioVU

1,826,189 probes (849,471 with rsid#:

Including newrs – 4865, Seq-rs – 1750, exm-rs - 7008)

1. Sexcheck
2. Relationship check
3. PCA to retain European ancestry individuals
4. Fetch rsids from UCSC genome ver. hg19 SNP150
5. Merge data using modified Illumina support probe rsid conversion file Nov 2017

1. Retain only single rsid for any probe that map to multiple rsids
2. Exclude non-rsid SNPs that map to rsid SNPs
3. Exclude probes that are not in autosomal and sex chromosomes (chromosomes labelled 0)

1,783,968 probes (1,778,177 with rsid#:

Including newrs – 662, Seq-rs – 1750, exm-rs - 1364)

1. retain autosomal SNPs with rsid

1,719,512 probes (Including newrs – 653, Seq-rs – 1184, exm-rs - 1327)

1. Impute using Michigan imputation server
2. Filter out SNPs be r^2>0.7
3. Reformate for PrediXcan/GWAS

PrediXcan

Suppl. Figure S3

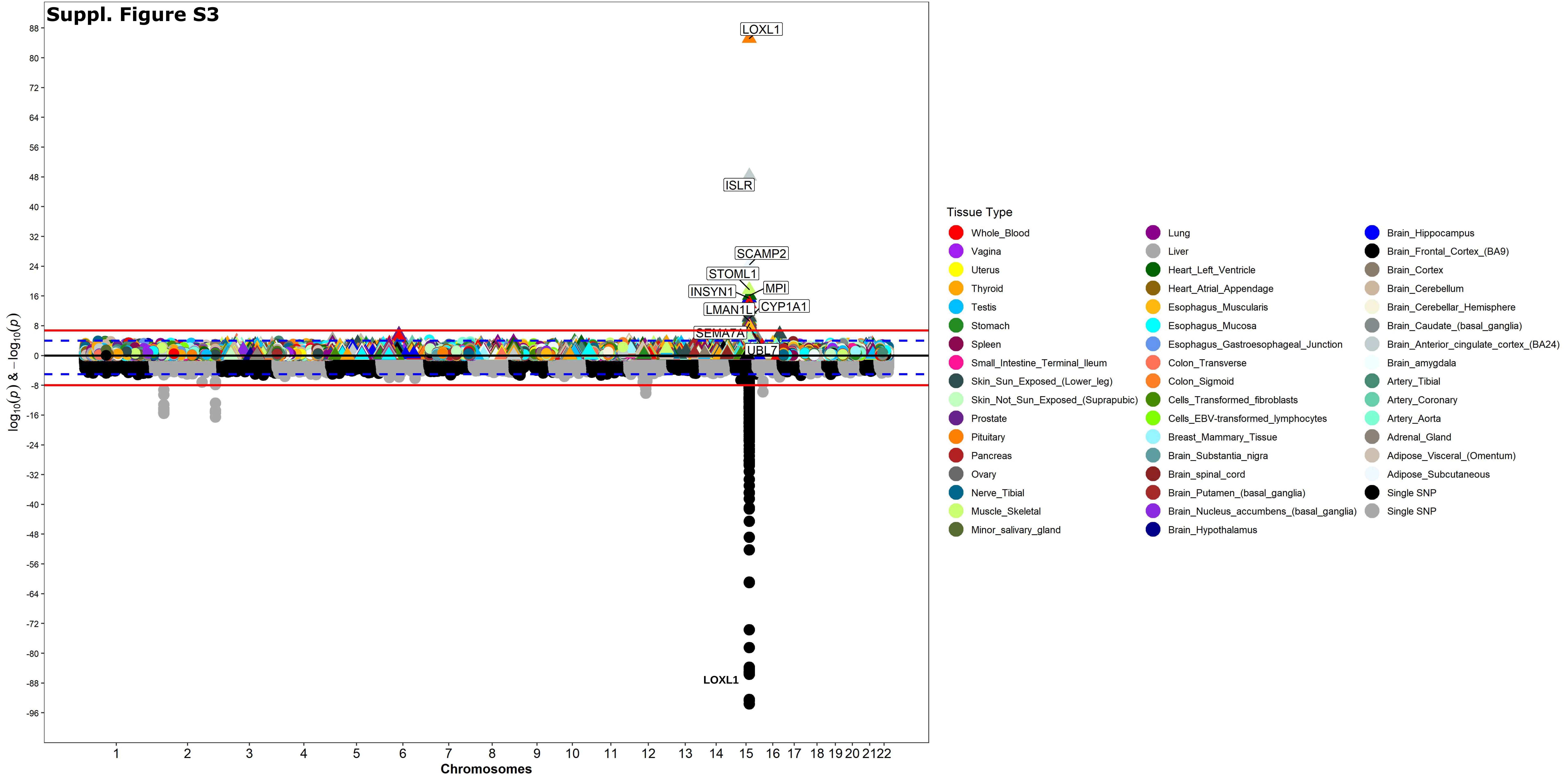

Suppl. Figure S4

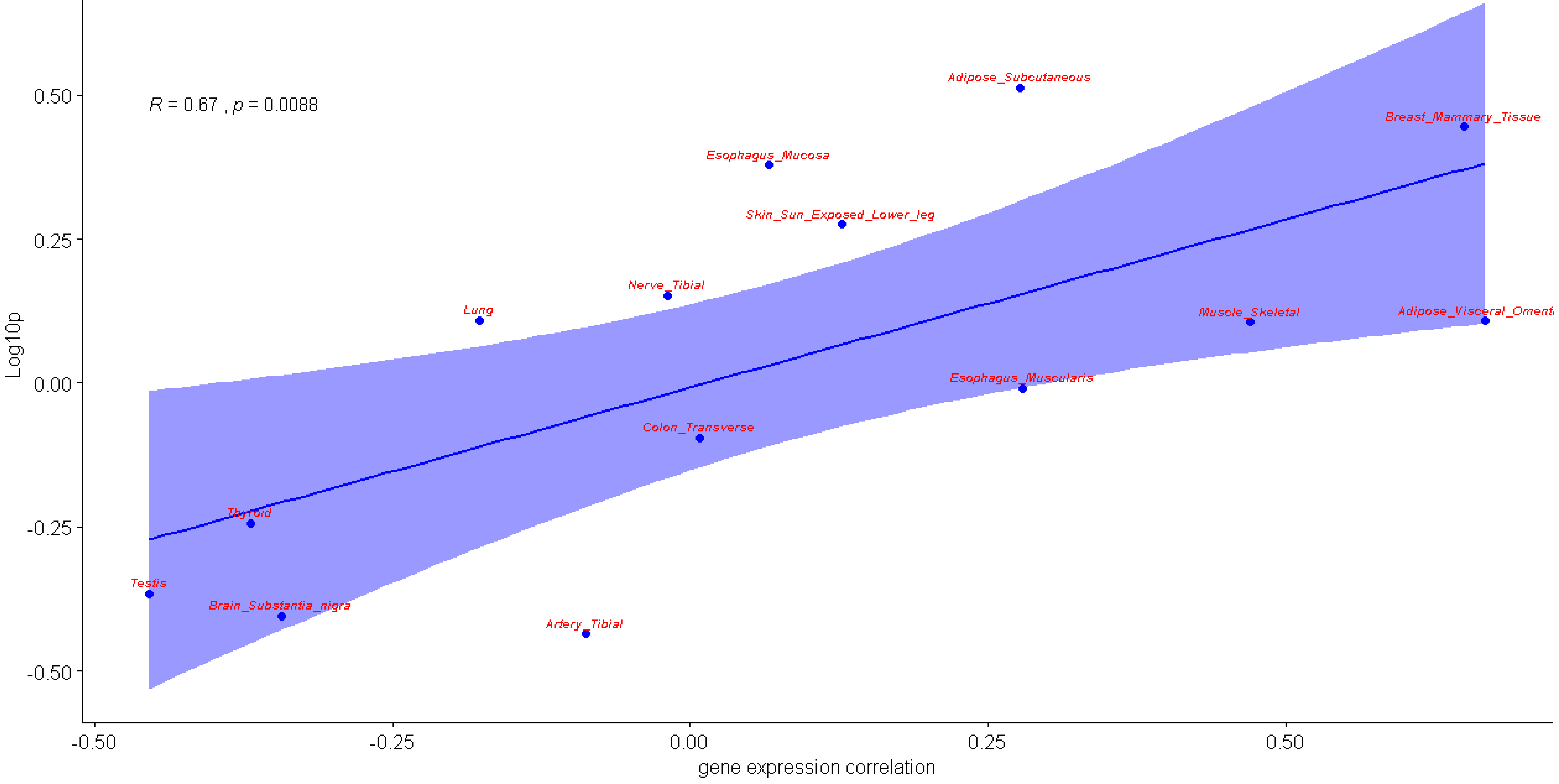

Suppl. Figure S5

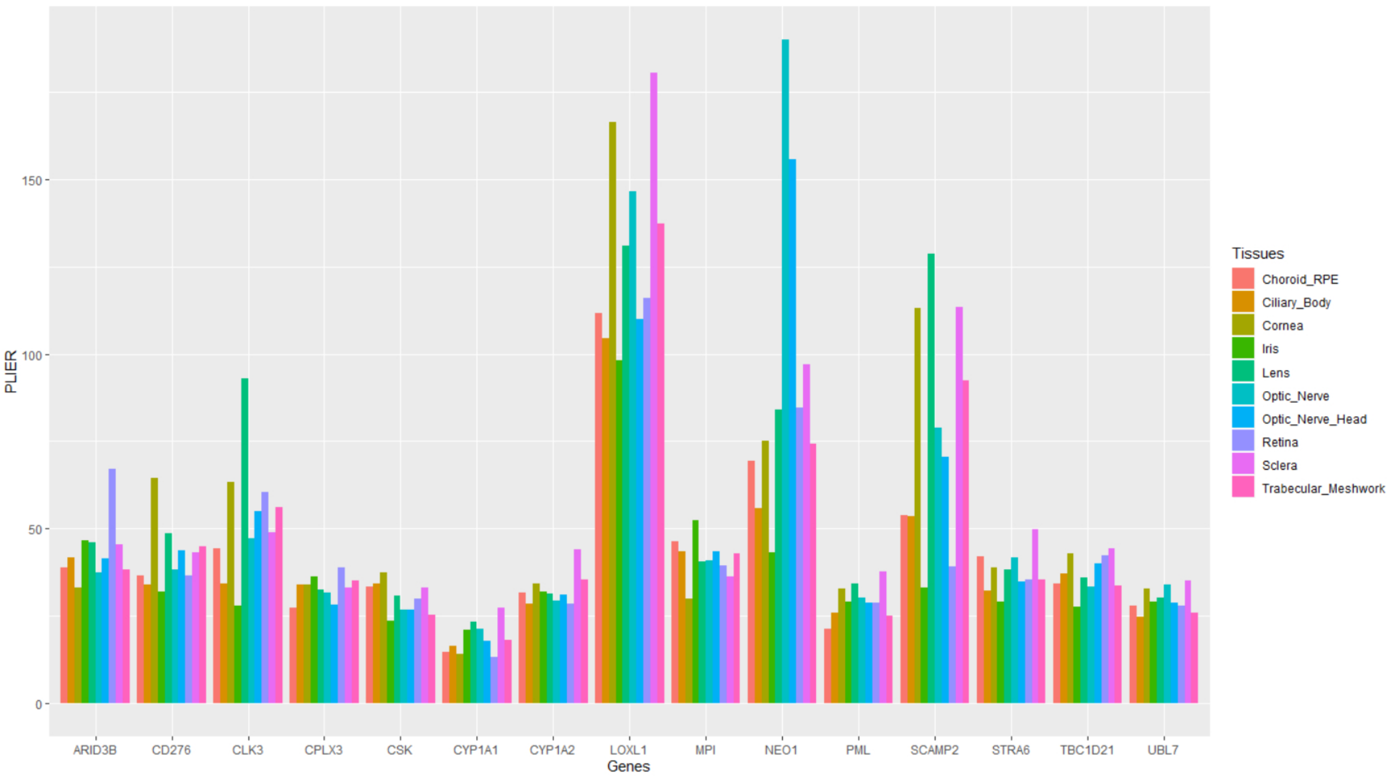

Suppl. Figure S6a

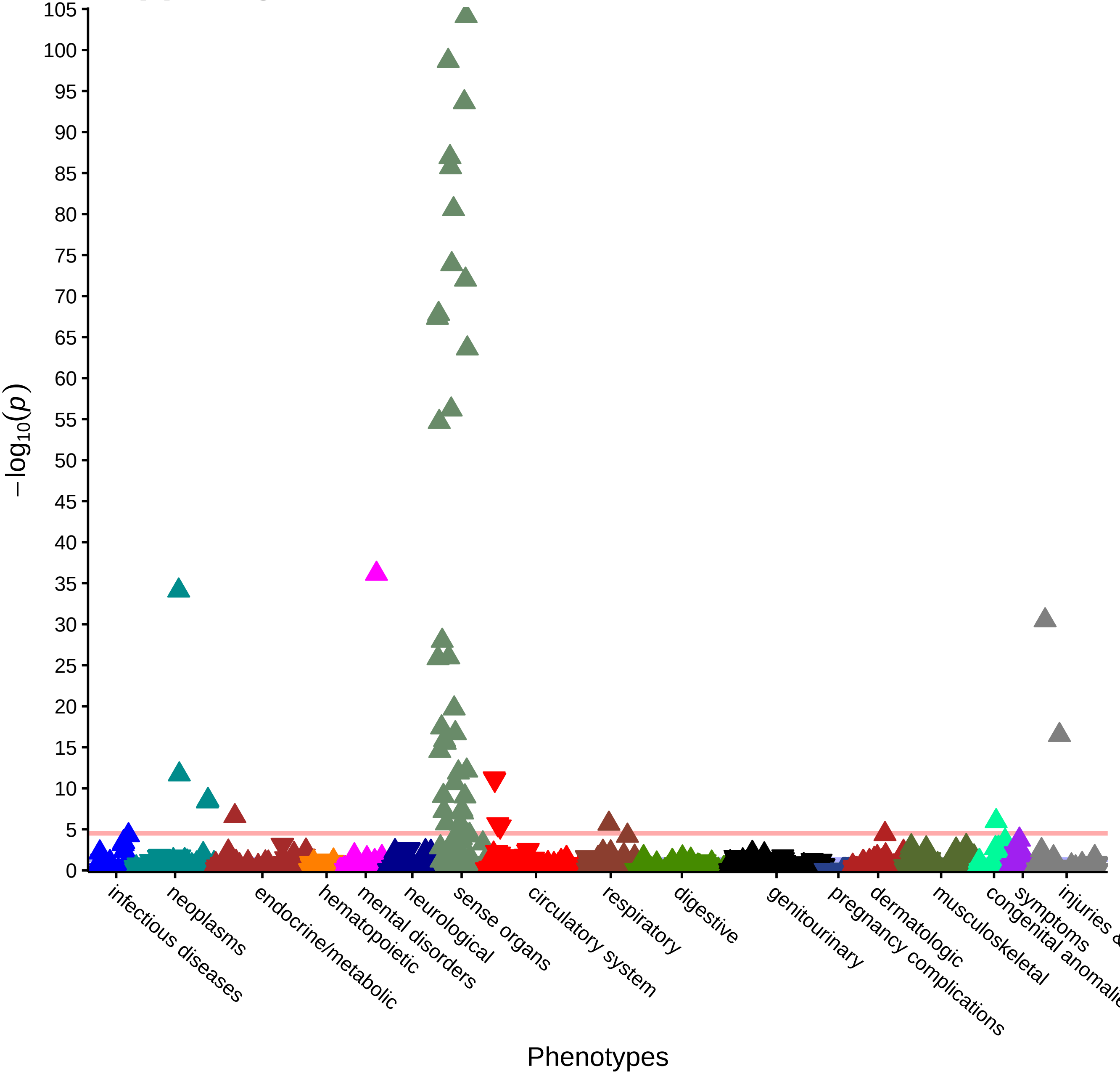

Suppl. Figure S6b

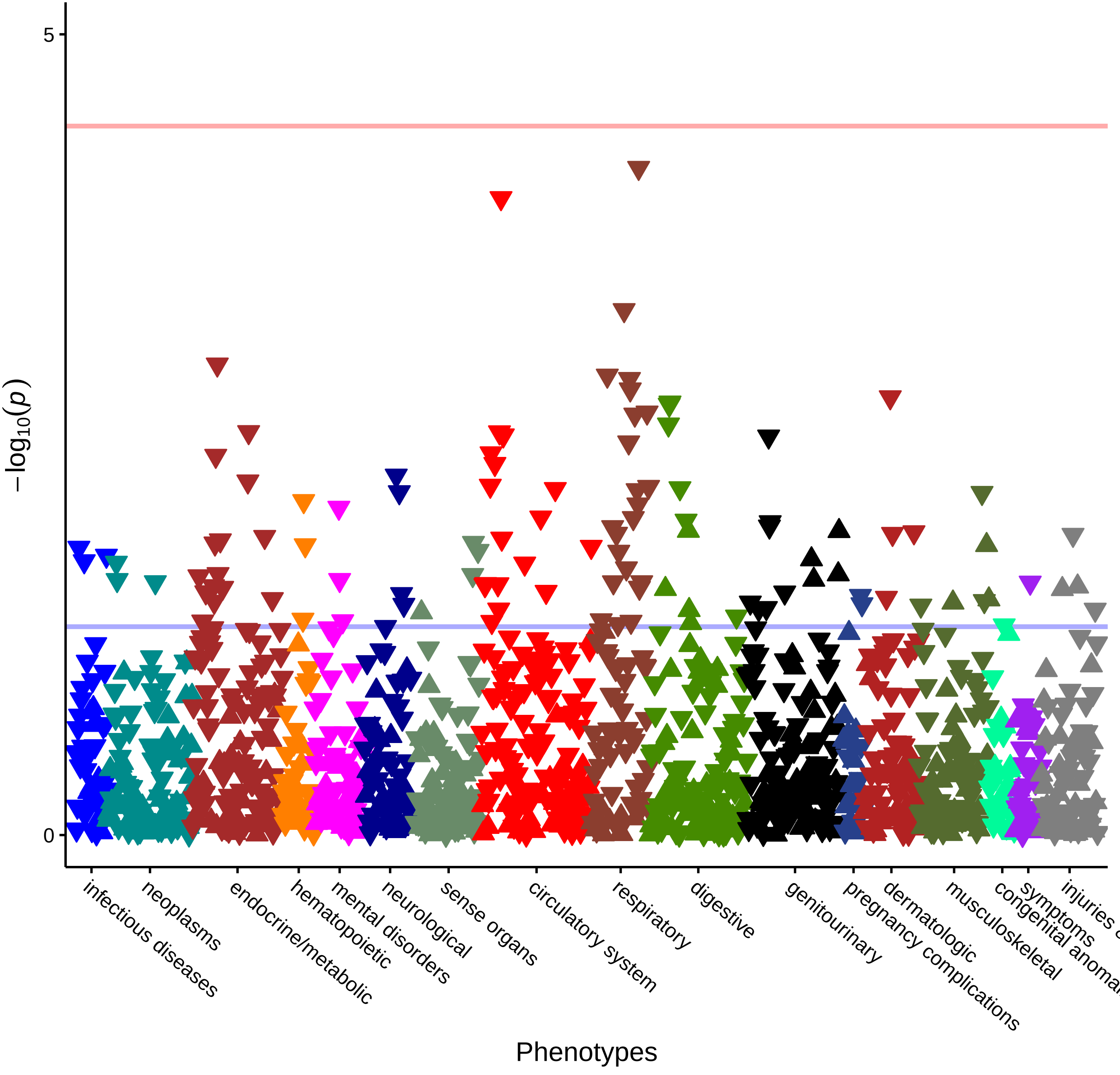

Supplement: Supplementary file 1 — Additional file 1. [file 12864_2023_9179_MOESM1_ESM.zip › Supplemental_figures_tables_combine.pdf]
